# Supplementary material for: Crystal Plasticity Parameter Optimization in Cyclically Deformed Electrodeposited Copper—A Machine Learning Approach
Source: Materials (Basel). 2024 Jul 9;17(14):3397. doi: 10.3390/ma17143397 (PMC11277840; doi:10.3390/ma17143397)
Supplement: Supplementary file 1 [file materials-17-03397-s001.zip › materials-3057381-supplementary.pdf]

Supplementary material for:

Crystal Plasticity Parameter Optimization in Cyclically Deformed Electrodeposited Copper—A Machine Learning Approach

by Karol Frydrych [1,2], Maciej Tomczak [1] and Stefanos Papanikolaou [1,*]

[1] NOMATEN Centre of Excellence, National Centre for Nuclear Research, Sołtana 7, 05-400 Otwock, Poland; karol.frydrych@ncbj.gov.pl (K.F.); maciej.tomczak@ncbj.gov.pl (M.T.)
[2] Institute of Fundamental Technological Research, Polish Academy of Sciences, Pawińskiego 5b, 02-106 Warsaw, Poland
[*] Correspondence: stefanos.papanikolaou@ncbj.gov.pl

Supplementary material

The supplementary material contains full set of results of the performed optimizations. For every optimal set of parameters, the result was verified by comparing the stress-strain curve obtained in simulation with this set of parameters against the reference one. As outlined in the article, the results were divided into 6 categories:

1. Very good or reasonable agreement of SS curves obtained using parameters optimized in both apporaches – Fig. S.1,
2. Disagreement in the first cycle and reasonable agreement of SS curves obtained using parameters optimized in both apporaches – Fig. S.2,
3. Reasonable agreement of SS curves obtained using parameters optimized in App 1 (lack of convergence for App 2 parameters) – Fig. S.3,
4. Reasonable agreement of SS curves obtained using parameters optimized in App 2 (lack of convergence for App 1 parameters) – Fig. S.4,
5. Striking disagreement or lack of convergence – Fig. S.5,
6. Lack of convergence for the optimized parameters in both approaches – Fig. S.6.

Tables S1-S6 present the corresponding values of isotropic and kinematic hardening param-eters. The results are discussed in the main part of the paper.

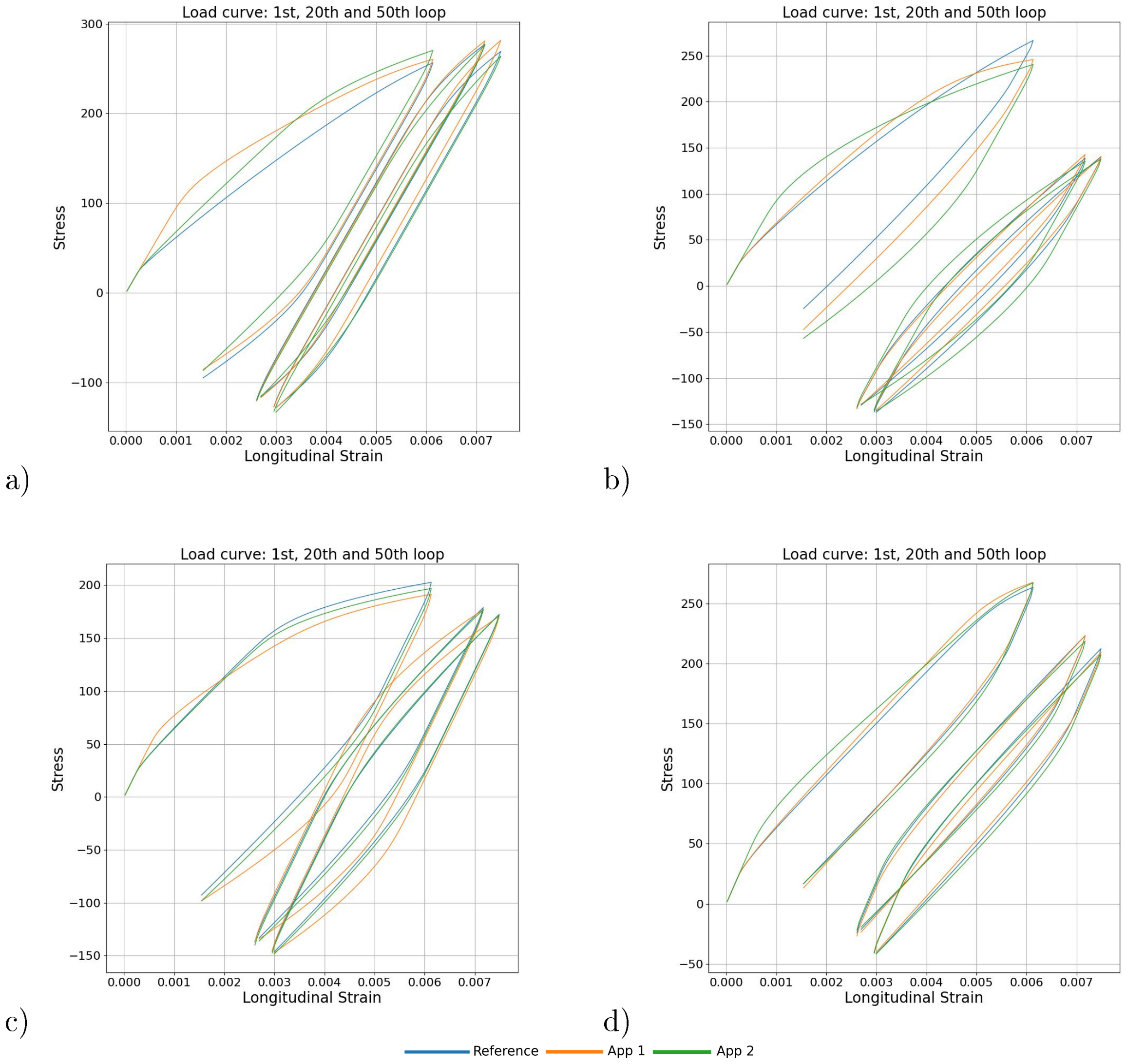

Figure S.1: The results of using neural networks for optimization: stress-strain curves obtained using the SEVPSC code for the arbitrary parameter set and its closest neighbours – Category 1: Very good or reasonable agreement of SS curves obtained using parameters optimized in both approaches).

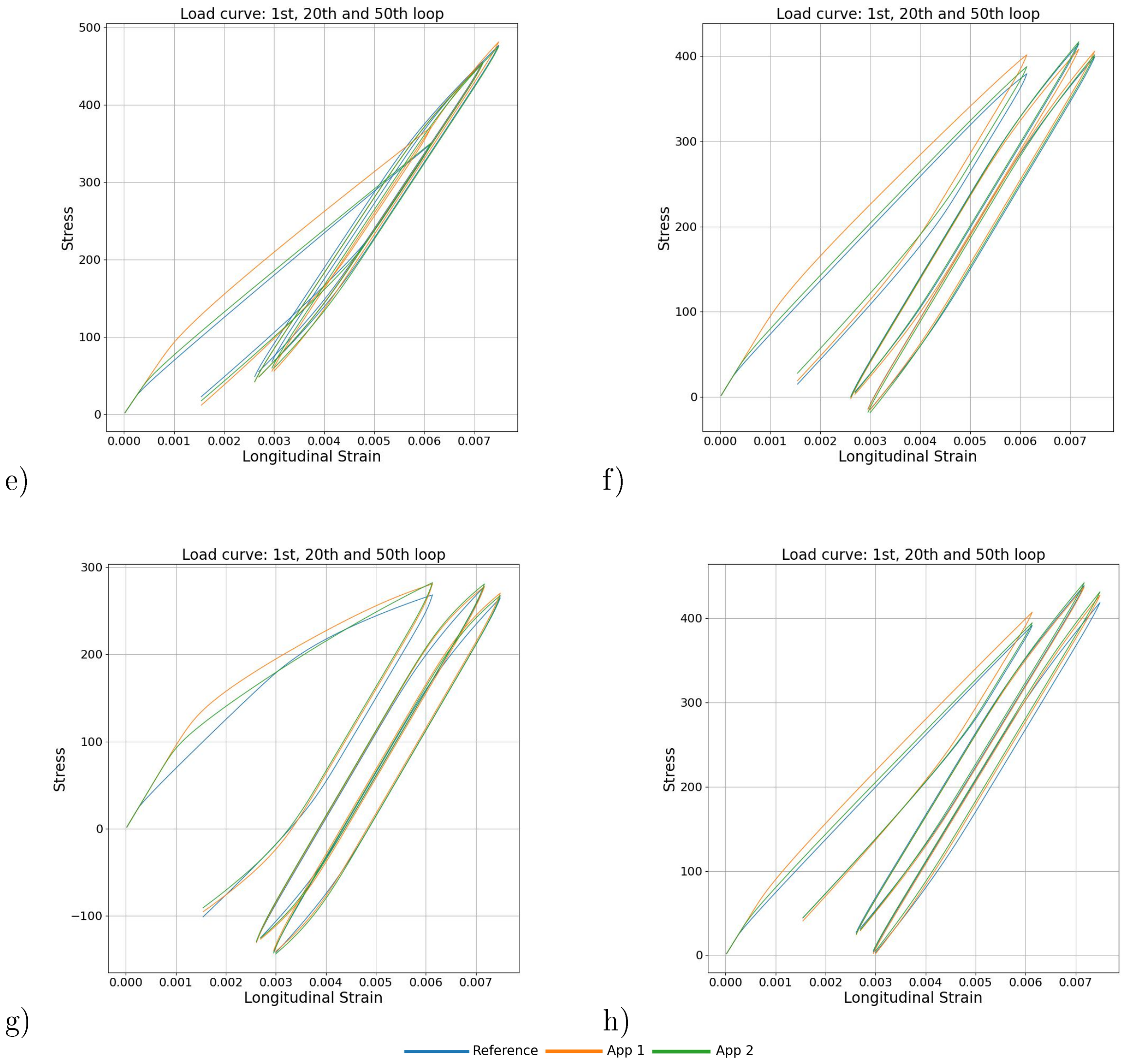

Figure S.1: Continued.

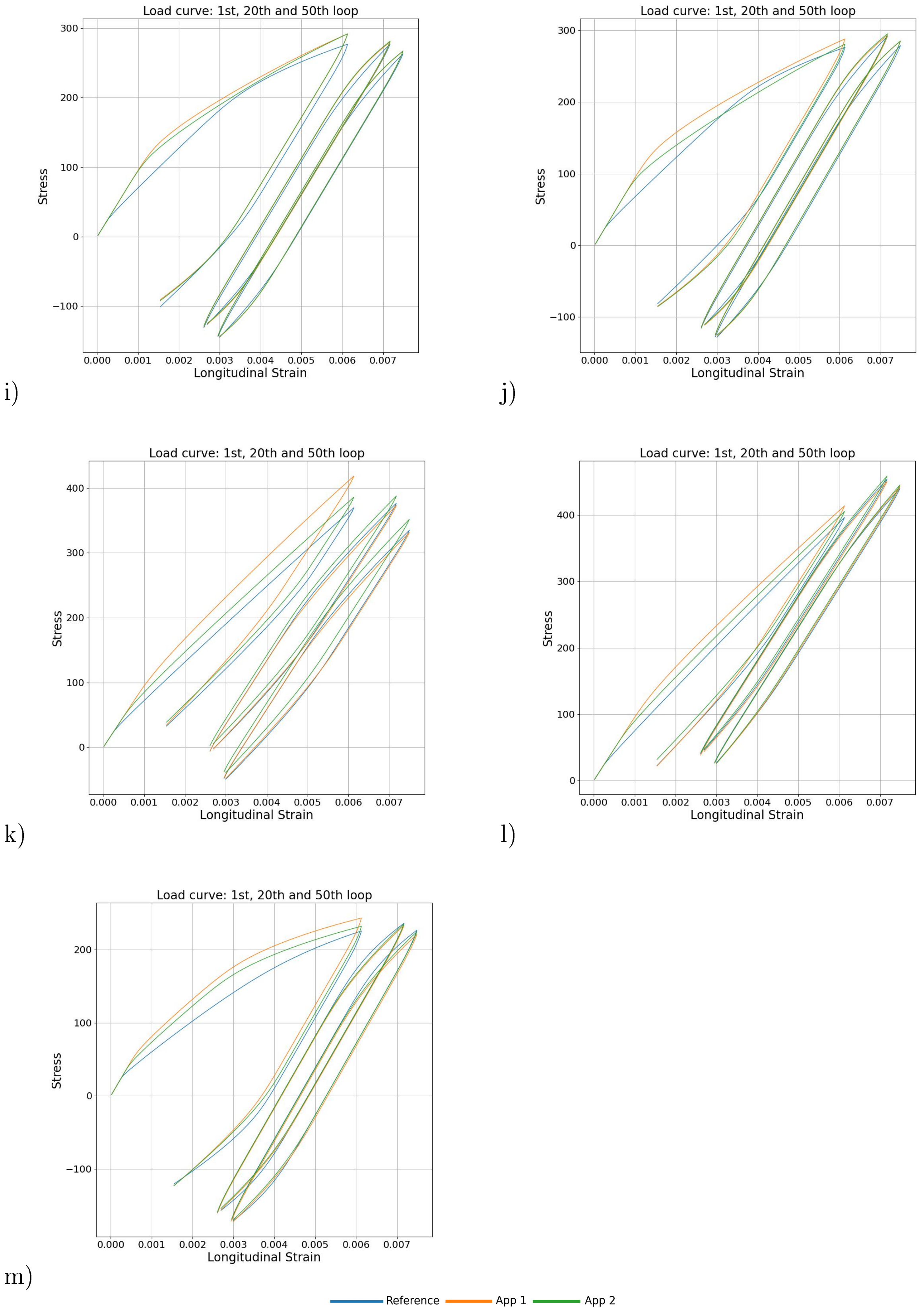

Figure S.1: Continued.

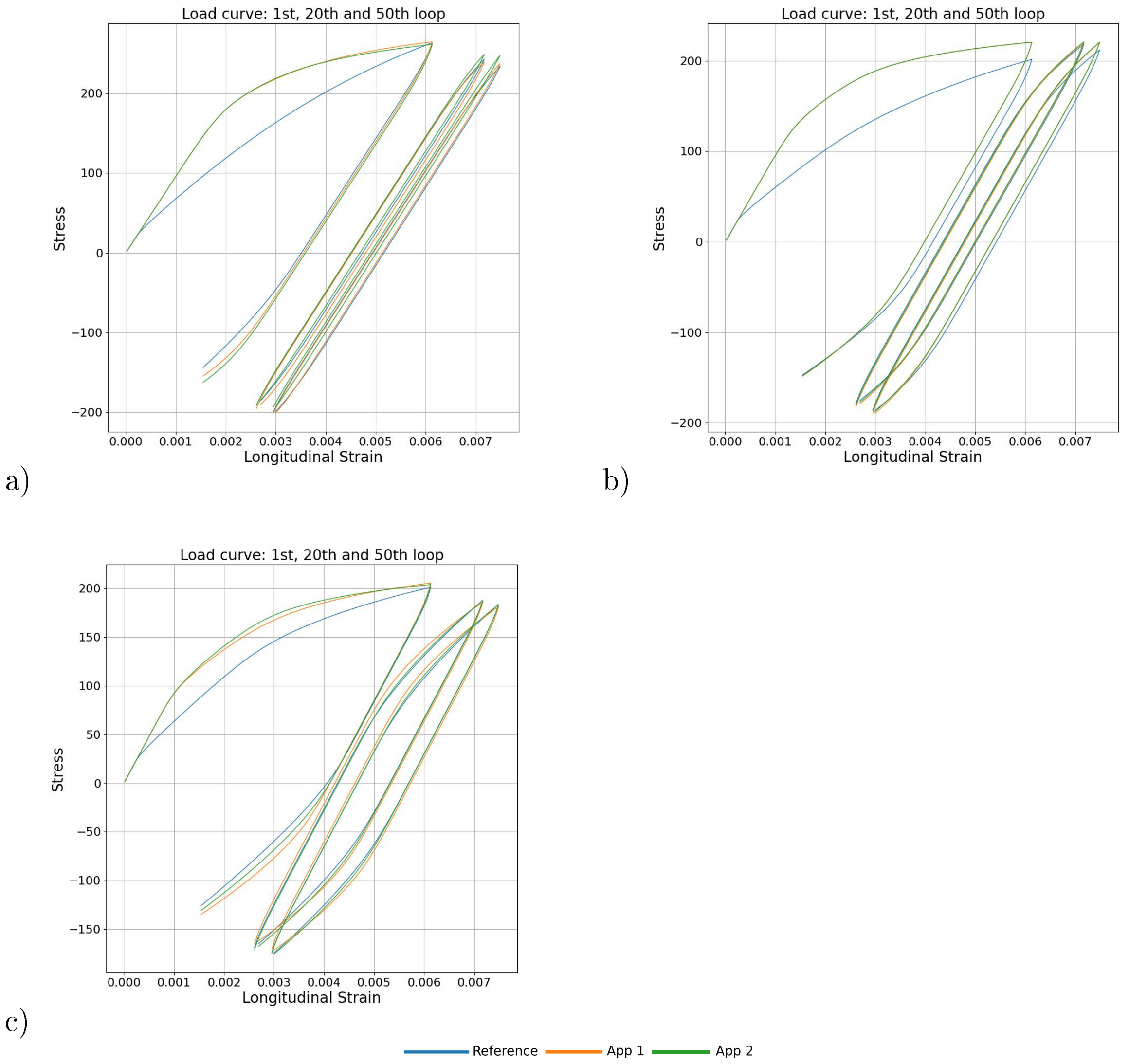

Figure S.2: The results of using neural networks for optimization: stress-strain curves obtained using the SEVPSC code for the arbitrary parameter set and its closest neighbours – Category 2: Disagreement in the first cycle and reasonable agreement of SS curves in further cycles obtained using parameters optimized in both approaches.

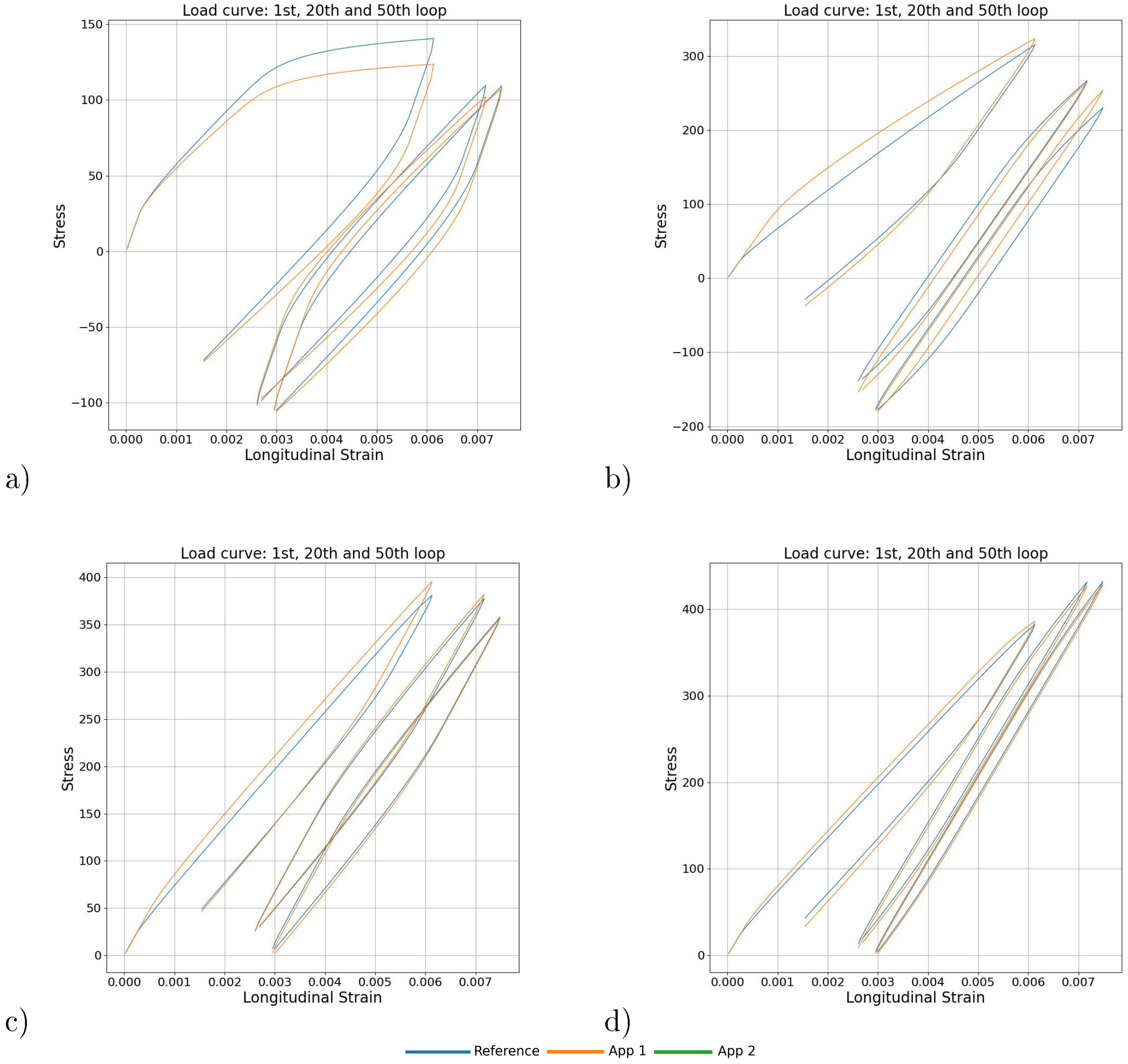

Figure S.3: The results of using neural networks for optimization: stress-strain curves obtained using the SEVPSC code for the arbitrary parameter set and its closest neighbours – Category 3: Reasonable agreement of SS curves obtained using parameters optimized in App 1 (lack of convergence for App 2 parameters).

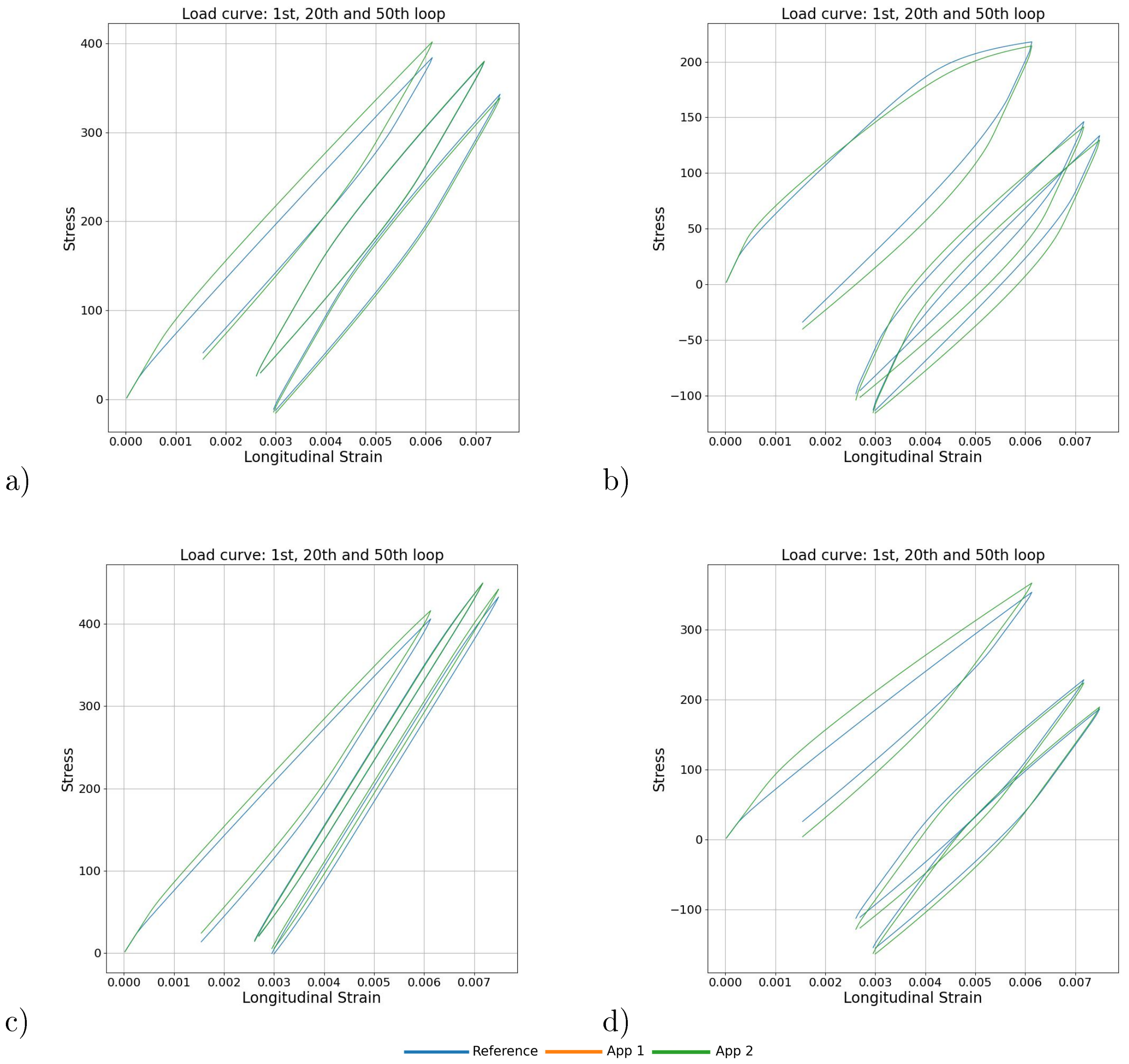

Figure S.4: The results of using neural networks for optimization: stress-strain curves obtained using the SEVPSC code for the arbitrary parameter set and its closest neighbours – Category 4: Reasonable agreement of SS curves obtained using parameters optimized in App 2 (lack of convergence for App 1 parameters).

	τ_c^0	τ_{sat}	h_1	h_b	h_k	h_{kb}	h_m
Reference	10.0	65.0	3.33	120.0	$1.67{\cdot}10^4$	$3.33{\cdot}10^2$	8.33
App. 1.	41.8	70.0	5.00	21.8	$1.82{\cdot}10^4$	$3.64{\cdot}10^2$	10.0
App. 2.	10.0	60.0	4.09	98.2	$4.55{\cdot}10^4$	$8.18{\cdot}10^2$	10.0
Reference	10.0	10.0	5.00	20.0	$6.67{\cdot}10^4$	$5.00{\cdot}10^2$	0.00
App. 1.	10.0	10.0	0.00	98.2	$7.27{\cdot}10^4$	$8.18{\cdot}10^2$	0.91
App. 2.	35.5	20.0	5.00	109.0	$3.64{\cdot}10^4$	$3.64{\cdot}10^2$	0.0
Reference	10.0	28.3	4.17	80.0	$5.00{\cdot}10^4$	$1.00{\cdot}10^3$	8.33
App. 1.	22.7	40.0	4.09	76.4	$1.82{\cdot}10^4$	$5.45{\cdot}10^2$	5.45
App. 2.	10.0	30.0	5.00	87.3	$4.55{\cdot}10^4$	$1.00{\cdot}10^3$	10.0
Reference	10.0	10.0	4.17	40.0	$5.00{\cdot}10^4$	$5.00{\cdot}10^2$	8.33
App. 1.	10.0	10.0	0.00	120.0	$5.45{\cdot}10^4$	$5.45{\cdot}10^2$	9.09
App. 2.	22.7	10.0	5.00	120.0	$4.55{\cdot}10^4$	$4.55{\cdot}10^2$	7.27
Reference	10.0	65.0	0.00	40.0	$6.67{\cdot}10^4$	$1.67{\cdot}10^2$	8.33
App. 1.	35.5	90.0	1.36	10.9	$6.36{\cdot}10^4$	$1.82{\cdot}10^2$	10.0
App. 2.	16.4	70.0	0.00	32.7	$6.36{\cdot}10^4$	90.9	10.0
Reference	10.0	65.0	2.50	80.0	$8.33{\cdot}10^4$	$6.67{\cdot}10^2$	10.0
App. 1.	41.8	80.0	1.82	10.9	$8.18{\cdot}10^4$	$6.36{\cdot}10^2$	9.09
App. 2.	16.4	70.0	0.00	43.6	$9.09{\cdot}10^4$	$6.36{\cdot}10^2$	5.45
Reference	10.0	65.0	0.00	100.0	$5.00{\cdot}10^4$	$1.00{\cdot}10^3$	10.0
App. 1.	48.2	70.0	5.00	43.6	$1.82{\cdot}10^4$	$3.64{\cdot}10^2$	5.45
App. 2.	35.5	70.0	5.00	87.3	$1.82{\cdot}10^4$	$2.73{\cdot}10^2$	2.73
Reference	10.0	65.0	4.17	40.0	$1.00{\cdot}10^5$	$5.00{\cdot}10^2$	3.33
App. 1.	29.1	90.0	4.09	10.9	$1.00{\cdot}10^5$	$4.55{\cdot}10^2$	2.73
App. 2.	16.4	80.0	1.36	21.8	$1.00{\cdot}10^5$	$4.55{\cdot}10^2$	2.73
Reference	10.0	65.0	4.17	120.0	$5.00{\cdot}10^4$	$1.00{\cdot}10^3$	8.33
App. 1.	48.2	70.0	5.00	65.5	$1.82{\cdot}10^4$	$2.73{\cdot}10^2$	2.73
App. 2.	41.8	70.0	4.55	98.2	$1.82{\cdot}10^4$	$2.73{\cdot}10^2$	2.73
Reference	10.0	65.0	0.00	80.0	$5.00{\cdot}10^4$	$8.33{\cdot}10^2$	8.33
App. 1.	48.2	70.0	5.00	43.6	$1.82{\cdot}10^4$	$2.73{\cdot}10^2$	4.55
App. 2.	35.5	70.0	2.73	76.4	$1.82{\cdot}10^4$	$2.73{\cdot}10^2$	4.55
Reference	10.0	46.7	3.33	60.0	$8.33{\cdot}10^4$	$3.33{\cdot}10^2$	1.67
App. 1.	41.8	50.0	0.46	0.0	$1.00{\cdot}10^5$	$5.45{\cdot}10^2$	2.73
App. 2.	22.7	50.0	0.00	21.8	$9.09{\cdot}10^4$	$3.64{\cdot}10^2$	1.82
Reference	10.0	65.0	4.17	120.0	$8.33{\cdot}10^4$	$5.00{\cdot}10^2$	6.67
App. 1.	48.2	70.0	1.82	21.8	$8.18{\cdot}10^4$	$4.55{\cdot}10^2$	5.45
App. 2.	29.1	70.0	0.00	43.6	$9.09{\cdot}10^4$	$3.64{\cdot}10^2$	2.73
Reference	10.0	65.0	4.17	100.0	$1.67{\cdot}10^4$	$5.00{\cdot}10^2$	3.33
App. 1.	22.7	60.0	2.27	98.2	$3.64{\cdot}10^4$	$1.00{\cdot}10^3$	5.45
App. 2.	16.4	60.0	5.00	87.3	$3.64{\cdot}10^4$	$1.00{\cdot}10^3$	6.36

Table S1: Reference parameters vs parameters that made LSTM generate closest matching curves – Category 1: Very good or reasonable agreement of SS curves obtained using parameters optimized in both approaches).

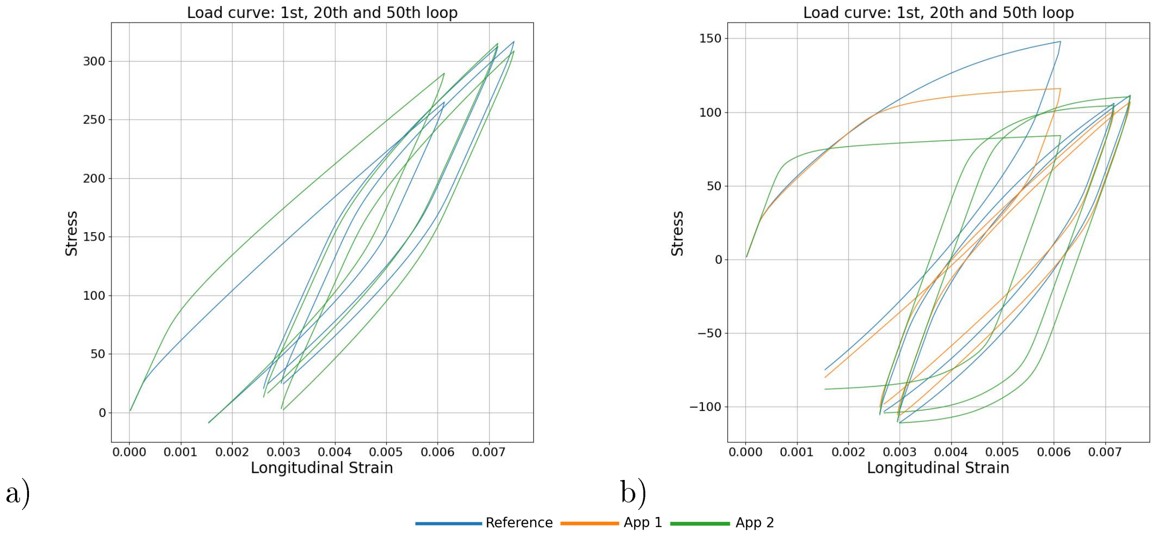

Figure S.5: The results of using neural networks for optimization: stress-strain curves obtained using the SEVPSC code for the arbitrary parameter set and its closest neighbours – Category 5: Striking disagreement or lack of convergence.

	τ_c^0	τ_{sat}	h_1	h_b	h_k	h_{kb}	h_m
Reference	10.0	83.3	0.83	120.0	$3.33 \cdot 10^4$	$1.00 \cdot 10^3$	0.00
App. 1.	67.3	100.0	5.00	10.9	$9.09 \cdot 10^3$	$5.45 \cdot 10^2$	0.91
App. 2.	67.3	120.0	0.46	10.9	$9.09 \cdot 10^3$	$8.18 \cdot 10^2$	3.64
Reference	10.0	65.0	5.00	100.0	$1.67 \cdot 10^4$	$8.33 \cdot 10^2$	3.33
App. 1.	48.2	70.0	3.64	21.8	$1.82 \cdot 10^4$	$1.00 \cdot 10^3$	4.55
App. 2.	48.2	70.0	0.00	21.8	$1.82 \cdot 10^4$	$1.00 \cdot 10^3$	5.45
Reference	10.0	46.7	4.17	100.0	$3.33 \cdot 10^4$	$1.00 \cdot 10^3$	3.33
App. 1.	35.5	50.0	3.18	98.2	$1.82 \cdot 10^4$	$8.18 \cdot 10^2$	4.55
App. 2.	35.5	50.0	0.00	32.7	$2.73 \cdot 10^4$	$1.00 \cdot 10^3$	3.64

Table S2: Reference parameters vs parameters that made LSTM generate closest matching curves – Category 2: Disagreement in the first cycle and reasonable agreement of SS curves in further cycles obtained using parameters optimized in both approaches.

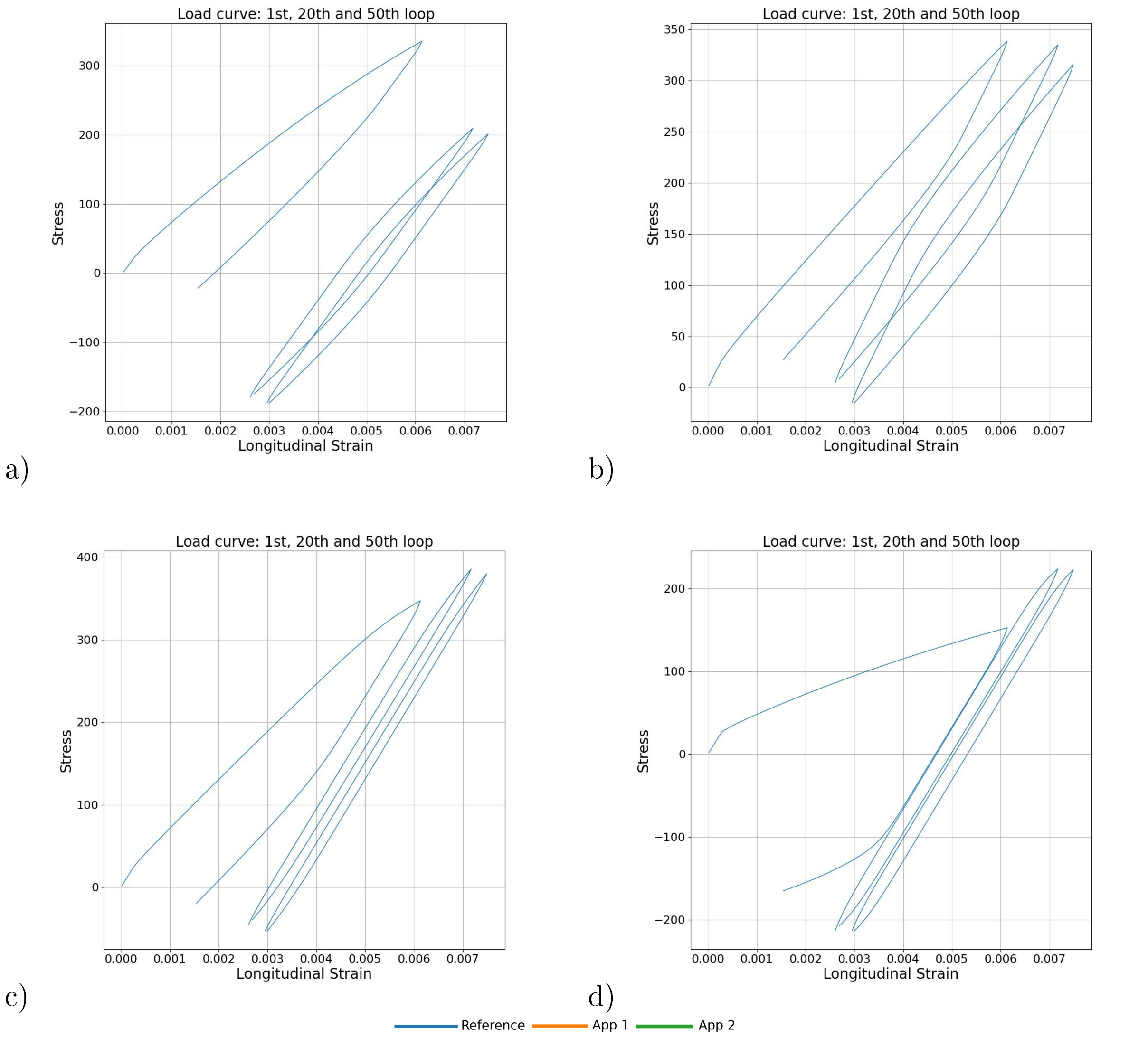

Figure S.6: The results of using neural networks for optimization: stress-strain curves obtained using the SEVPSC code for the arbitrary parameter set and its closest neighbours – Category 6: Lack of convergence for the optimized parameters in both approaches.

	τ_c^0	τ_{sat}	h_1	h_b	h_k	h_{kb}	h_m
Reference	10.0	10.0	1.67	120.0	$3.33{\cdot}10^4$	$8.33{\cdot}10^2$	8.33
App. 1.	10.0	10.0	5.00	21.8	$2.73{\cdot}10^4$	$8.18{\cdot}10^2$	7.27
App. 2.	16.4	10.0	5.00	21.8	$2.73{\cdot}10^4$	$7.27{\cdot}10^2$	3.64
Reference	10.0	65.0	0.83	60.0	$5.00{\cdot}10^4$	$1.67{\cdot}10^2$	0.00
App. 1.	35.5	100.0	2.27	10.9	$4.55{\cdot}10^4$	$1.82{\cdot}10^2$	0.00
App. 2.	29.1	70.0	0.46	21.8	$6.36{\cdot}10^4$	$4.55{\cdot}10^2$	90.9
Reference	10.0	28.3	0.00	80.0	$1.00{\cdot}10^5$	$6.67{\cdot}10^2$	10.0
App. 1.	22.7	30.0	0.00	76.4	$1.00{\cdot}10^5$	$6.36{\cdot}10^2$	7.27
App. 2.	22.7	30.0	0.00	21.8	$1.00{\cdot}10^5$	$6.36{\cdot}10^2$	7.27
Reference	10.0	83.3	0.83	20.0	$1.00{\cdot}10^5$	$6.67{\cdot}10^2$	6.67
App. 1.	16.4	80.0	1.36	21.8	$1.00{\cdot}10^5$	$7.27{\cdot}10^2$	9.09
App. 2.	16.4	90.0	5.00	21.8	$9.09{\cdot}10^4$	$6.36{\cdot}10^2$	8.18

Table S3: Reference parameters vs parameters that made LSTM generate closest match-ing curves Category 3: Reasonable agreement of SS curves obtained using parameters optimized in App 1 (lack of convergence for App 2 parameters).

	τ_c^0	τ_{sat}	h_1	h_b	h_k	h_{kb}	h_m
Reference	10.0	28.3	5.00	80.0	$1.00{\cdot}10^5$	$5.00{\cdot}10^2$	3.33
App. 1.	41.8	20.0	1.82	21.8	$1.00{\cdot}10^5$	$4.55{\cdot}10^2$	2.73
App. 2.	29.1	120.0	1.36	0.00	$1.00{\cdot}10^5$	$4.55{\cdot}10^2$	2.73
Reference	10.0	10.0	1.67	100.0	$5.00{\cdot}10^4$	$6.67{\cdot}10^2$	3.33
App. 1.	22.7	10.0	5.00	43.6	$3.64{\cdot}10^4$	$4.55{\cdot}10^2$	1.82
App. 2.	16.4	60.0	0.00	0.00	$3.64{\cdot}10^4$	$5.45{\cdot}10^2$	2.73
Reference	10.0	83.3	5.00	120.0	$8.33{\cdot}10^4$	$5.00{\cdot}10^2$	3.33
App. 1.	35.5	90.0	0.00	32.7	$1.00{\cdot}10^5$	$7.27{\cdot}10^2$	5.45
App. 2.	22.7	90.0	0.00	65.5	$1.00{\cdot}10^5$	$7.27{\cdot}10^2$	5.45
Reference	10.0	28.3	0.00	80.0	$8.33{\cdot}10^4$	$1.67{\cdot}10^2$	0.00
App. 1.	35.5	20.0	0.00	43.6	$9.09{\cdot}10^4$	$4.55{\cdot}10^2$	0.91
App. 2.	35.5	20.0	0.00	0.0	$7.27{\cdot}10^4$	$1.82{\cdot}10^2$	0.0

Table S4: Reference parameters vs parameters that made LSTM generate closest match-ing curves Category 4: Reasonable agreement of SS curves obtained using parameters optimized in App 2 (lack of convergence for App 1 parameters).

	τ_c^0	τ_{sat}	h_1	h_b	h_k	h_{kb}	h_m
Reference	10.0	28.3	0.83	120.0	$3.33{\cdot}10^4$	$1.67{\cdot}10^2$	5.00
App. 1.	35.5	20.0	3.18	21.8	$3.64{\cdot}10^4$	$2.73{\cdot}10^2$	10.0
App. 2.	29.1	20.0	4.55	0.0	$3.64{\cdot}10^4$	$1.82{\cdot}10^2$	3.64
Reference	10.0	10.0	5.00	120.0	$3.33{\cdot}10^4$	$6.67{\cdot}10^2$	0.00
App. 1.	10.0	10.0	5.00	21.8	$2.73{\cdot}10^4$	$9.09{\cdot}10^2$	8.18
App. 2.	22.7	30.0	5.00	32.7	0.0	0.0	0.0

Table S5: Reference parameters vs parameters that made LSTM generate closest matching curves – Category 5: Striking disagreement or lack of convergence.

	τ_c^0	τ_{sat}	h_1	h_b	h_k	h_{kb}	h_m
Category 6							
Reference	10.0	46.7	2.50	40.0	$1.00 \cdot 10^5$	$6.67 \cdot 10^2$	0.00
App. 1.	10.0	50.0	2.73	120.0	$7.27 \cdot 10^4$	$5.45 \cdot 10^2$	0.00
App. 2.	16.4	50.0	5.00	65.5	$7.27 \cdot 10^4$	$5.45 \cdot 10^2$	0.0
Reference	10.0	28.3	1.67	100.0	$6.67 \cdot 10^4$	$5.00 \cdot 10^2$	8.33
App. 1.	10.0	20.0	0.00	43.6	$1.00 \cdot 10^5$	$7.27 \cdot 10^2$	10.0
App. 2.	10.0	20.0	2.27	21.8	$1.00 \cdot 10^5$	$7.27 \cdot 10^2$	10.0
Reference	10.0	83.3	5.00	60.0	$6.67 \cdot 10^4$	$6.67 \cdot 10^2$	6.67
App. 1.	29.1	90.0	0.00	32.7	$7.27 \cdot 10^4$	$8.18 \cdot 10^2$	10.0
App. 2.	16.4	90.0	3.64	43.6	$7.27 \cdot 10^4$	$7.27 \cdot 10^2$	6.36
Reference	10.0	83.3	1.67	60.0	0.00	1000.00	0.00
App. 1.	35.5	90.0	2.73	43.6	0.0	1000.00	4.55
App. 2.	22.7	90.0	0.91	54.6	0.0	727.27	0.0

Table S6: Reference parameters vs parameters that made LSTM generate closest matching curves – Category 6: Lack of convergence for the optimized parameters in both approaches.
